# Incidence and prevalence of traumatic spinal cord injury in Canada using health administrative data

**DOI:** 10.3389/fneur.2023.1201025

**Published:** 2023-07-24

**Authors:** Nancy P. Thorogood, Vanessa K. Noonan, Xiaozhi Chen, Nader Fallah, Suzanne Humphreys, Nicolas Dea, Brian K. Kwon, Marcel F. Dvorak

**Affiliations:** ^1^Praxis Spinal Cord Institute, Vancouver, BC, Canada; ^2^Department of Medicine, University of British Columbia, Vancouver, BC, Canada; ^3^Combined Neurosurgery and Orthopaedic Spine Program, University of British Columbia, Vancouver, BC, Canada; ^4^International Collaboration on Repair Discoveries (ICORD), University of British Columbia, Vancouver, BC, Canada

**Keywords:** spinal cord injury, epidemiology, incidence, prevalence, routinely collected health data

## Abstract

**Introduction:**

Incidence and prevalence data are needed for the planning, funding, delivery and evaluation of injury prevention and health care programs. The objective of this study was to estimate the Canadian traumatic spinal cord injury (TSCI) incidence, prevalence and trends over time using national-level health administrative data.

**Methods:**

ICD-10 CA codes were used to identify the cases for the hospital admission and discharge incidence rates of TSCI in Canada from 2005 to 2016. Provincial estimates were calculated using the location of the admitting facility. Age and sex-specific incidence rates were set to the 2015/2016 rates for the 2017 to 2019 estimates. Annual incidence rates were used as input for the prevalence model that applied annual survivorship rates derived from life expectancy data.

**Results:**

For 2019, it was estimated that there were 1,199 cases (32.0 per million) of TSCI admitted to hospitals, with 123 (10% of admissions) in-hospital deaths and 1,076 people with TSCI (28.7 per million) were discharged in Canada. The estimated number of people living with TSCI was 30,239 (804/million); 15,533 (52%) with paraplegia and 14,706 (48%) with tetraplegia. Trends included an increase in the number of people injured each year from 874 to 1,199 incident cases (37%), an older average age at injury rising from 46.6 years to 54.3 years and a larger proportion over the age of 65 changing from 22 to 38%, during the 15-year time frame.

**Conclusion:**

This study provides a standard method for calculating the incidence and prevalence of TSCI in Canada using national-level health administrative data. The estimates are conservative based on the limitations of the data but represent a large Canadian sample over 15 years, which highlight national trends. An increasing number of TSCI cases among the elderly population due to falls reported in this study can inform health care planning, prevention strategies, and future research.

## Introduction

Spinal cord injury (SCI) is a relatively uncommon injury compared to other health conditions, however, its occurrence has profound long-lasting personal, social and financial impacts on individuals, families, the health care system and society. Estimates of incidence and prevalence are needed for the planning, funding, delivery and evaluation of injury prevention and health care programs.

Incidence and prevalence rates vary based on data sources used and the way data are collected and validated. Data collected for administrative or billing purposes in a health care system are referred to as health administrative data (1). In Canada, a record is created for every hospital visit upon discharge and contains demographic, administrative and some clinical data (2). Diagnoses and procedures are coded using the International Statistical Classification of Diseases and Related Health Problems (ICD) codes and these data are managed nationally by the Canadian Institute of Health Information (CIHI). While health administrative data can describe a population, they lack detailed clinical and neurological data. Acknowledging its limitations, health administrative data have been utilized to estimate the incidence of traumatic spinal cord injury (TSCI) in several countries (3–6).

Health administrative data have previously been used to estimate the incidence of TSCI within specific regions or single provinces of Canada, such as Ontario reporting 21 to 49/million (7–9), Alberta reporting 52/million (10), British Columbia reporting 36/million (11), and Manitoba reporting 17 to 26/million (12). We previously estimated the Canadian TSCI incidence to be 41/million and the prevalence as 1,300/million by applying health administrative data from a single province (Alberta) to the Canadian population in 2010 (13). There are subtle differences in case ascertainment among these studies (e.g., age restrictions, inclusion or exclusion of those who do not survive initial hospital stay, different TSCI classification). A standard method for estimating TSCI incidence described in our methods may advance the field and enable accurate comparisons among regions and over time. We therefore wanted to update these estimates with national-level health administrative data, provide provincial estimates and examine trends over time.

The objective of this study was to estimate the Canadian TSCI incidence, prevalence and trends over time using national-level health administrative data from January 2005 to December 2016 and estimate to 2019. The results were compared to previous estimates and other countries that have published TSCI incidence rates using administrative data.

## Materials and methods

### Data specification

Data were requested from CIHI (14), for the National Trauma Registry (NTR) (15) from April 1, 2004 to March 31, 2011 and for the Discharge Abstract Database (DAD) (2) from April 1, 2011 to March 31, 2017 (for a schematic, see Supplementary Figure 1). The time periods reflect when the required data were available and coded with ICD-10 CA (Tenth Revision, Canada). The NTR ended in 2011 and the DAD included the data variables from NTR’s minimal data set. These data sources include all provinces and territories in Canada, except Québec and are collected in the same manner. Cases of TSCI were selected on the criteria of an ICD-10 CA TSCI code (see codes and descriptions in Table 1), an external cause of injury code and an acute facility code.

**Table 1:** ICD-10 CA codes used to identify traumatic spinal cord injury cases.

ICD-10 code	Code description
S14.0	Concussion and oedema of cervical spinal cord
S14.10	Complete lesion of cervical spinal cord
S14.11	Central cord lesion of cervical spinal cord
S14.12	Anterior cord syndrome of cervical spinal cord
S14.13	Posterior cord syndrome of cervical spinal cord
S14.18	Other injuries of cervical spinal cord
S14.19	Unspecified lesion of cervical spinal cord
S24.0	Concussion and oedema of thoracic spinal cord
S24.10	Complete lesion of thoracic spinal cord
S24.11	Central cord lesion of thoracic spinal cord
S24.12	Anterior cord syndrome of thoracic spinal cord
S24.13	Posterior cord syndrome of thoracic spinal cord
S24.18	Other injuries of thoracic spinal cord
S24.19	Unspecified lesion of thoracic spinal cord
S34.0	Concussion and oedema of lumbar spinal cord
S34.10	Complete lesion of lumbar spinal cord
S34.11	Central cord lesion of lumbar spinal cord
S34.12	Anterior cord syndrome of lumbar spinal cord
S34.13	Posterior cord syndrome of lumbar spinal cord
S34.18	Other injuries of lumbar spinal cord
S34.19	Unspecified lesion of lumbar spinal cord
S34.30	Laceration of cauda equina
S34.38	Other and unspecified injury of cauda equina
T06.0	Injuries of brain and cranial nerves with injuries of nerves and spinal cord at neck level
T06.1	Injuries of nerves and spinal cord involving other multiple body regions

The NTR excluded external causes of injury resulting from complications, misadventures, adverse incidents/reactions from surgical care (for associated ICD-10 CA codes see Supplementary Table 1), therefore these cases were excluded from the DAD extract and all subsequent estimates based on this data. To ensure confidentiality, cells were suppressed when values were less than 5 observations. Years were combined into pairs for the 2005 to 2016 period to avoid a large number of suppressed cells for tabulations of five-year age groups by sex.

### Procedure for estimating TSCI incidence

**Figure 1:**
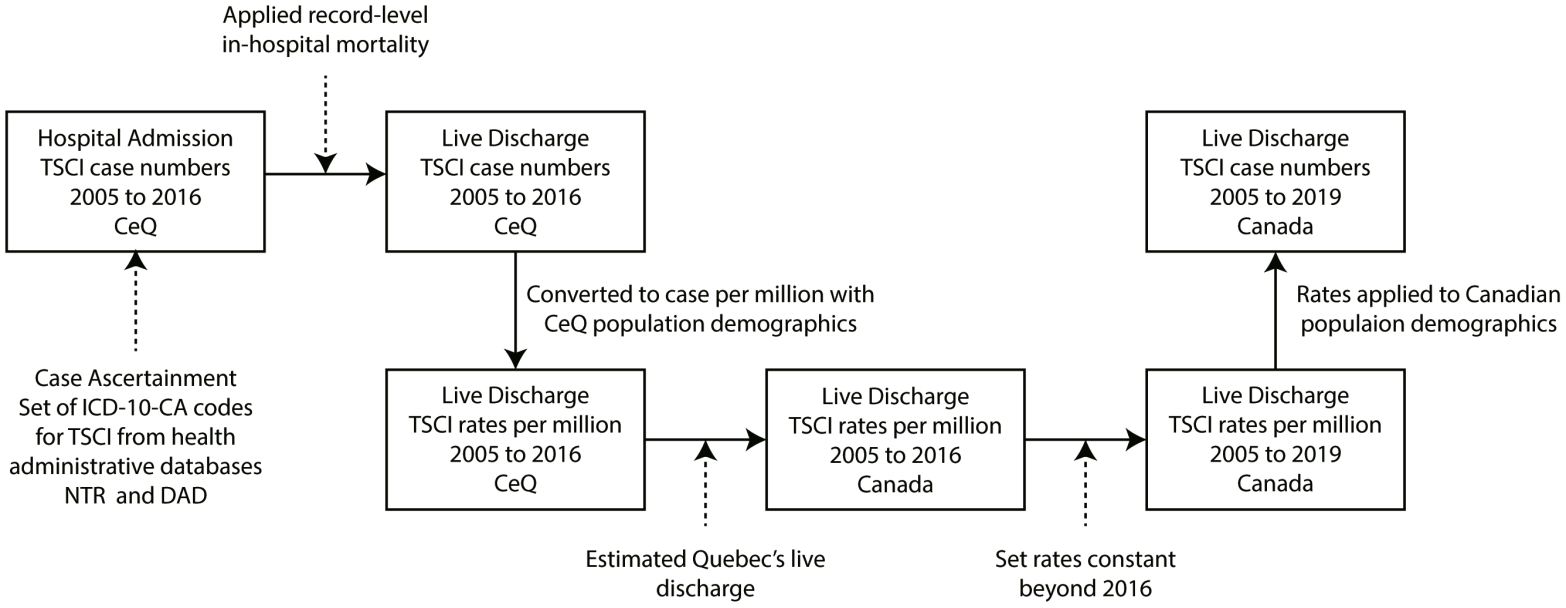
Process map of traumatic spinal cord injury (TSCI) incidence estimation methodology. All TSCI case numbers and rates were calculated by five-year age- and sex-specific groups. ICD-10-CA, International Classification of Disease, Version 10, Canadian revision; TSCI, traumatic spinal cord injury; CeQ, Canada excluding Québec; DAD, Discharge Abstract Database; NTR, National Trauma Registry.

Data were tabulated by unique identifiers and indexed by date to count all unique individuals by age and sex that had an ICD-10 code from Table 1 in the diagnosis variable (for the aggregated data see Supplementary Table 2). The discharge incidence represents the unique individuals that survived the initial hospital stay and were discharged from an acute facility. Survival and mortality were defined by the discharge disposition data variable. The methodology developed to calculate the admission and discharge incidences of TSCI is shown in Figure 1. The discharge rates for Canada, except Québec, were calculated by subtracting the number of in-hospital deaths from the number of TSCI admission cases and dividing by the corresponding population, from Statistics Canada’s annual population estimates. The provincial admission incidence estimates were based on the location of the admitting facility and the corresponding provincial population from Statistics Canada (16).

To derive national rates that included an estimation for Québec, we accessed Injury and Trauma Emergency Department and Hospitalization Statistics reports that contained data on all injury and trauma visits to the emergency department and hospitalizations in acute care hospitals in Canadian provinces, including Québec, from 2014 to 2018 (17). Provincial hospitalizations and external cause of injury data were used as an indication of how rates of TSCI compare among provinces in Canada. Specifically, provincial variances were applied to Québec’s population to estimate Québec’s provincial TSCI incidence rates. These rates were incorporated into the rates that excluded Québec to estimate the national rates for the period 2005 to 2016. To calculate the rates for 2017 to 2019, it was assumed that the 2015 to 2016 period rates were stable for these 3 years.

The national annual rates were then multiplied by the Canadian population (16) for each year to estimate annual discharge TSCI incidence case numbers by age group and sex. Using these annual case numbers, in-hospital mortality relative to discharge ratios were calculated from the health administrative data, which were then used to estimate national annual admission case numbers.

### Procedure for estimating TSCI prevalence

**Figure 2:**
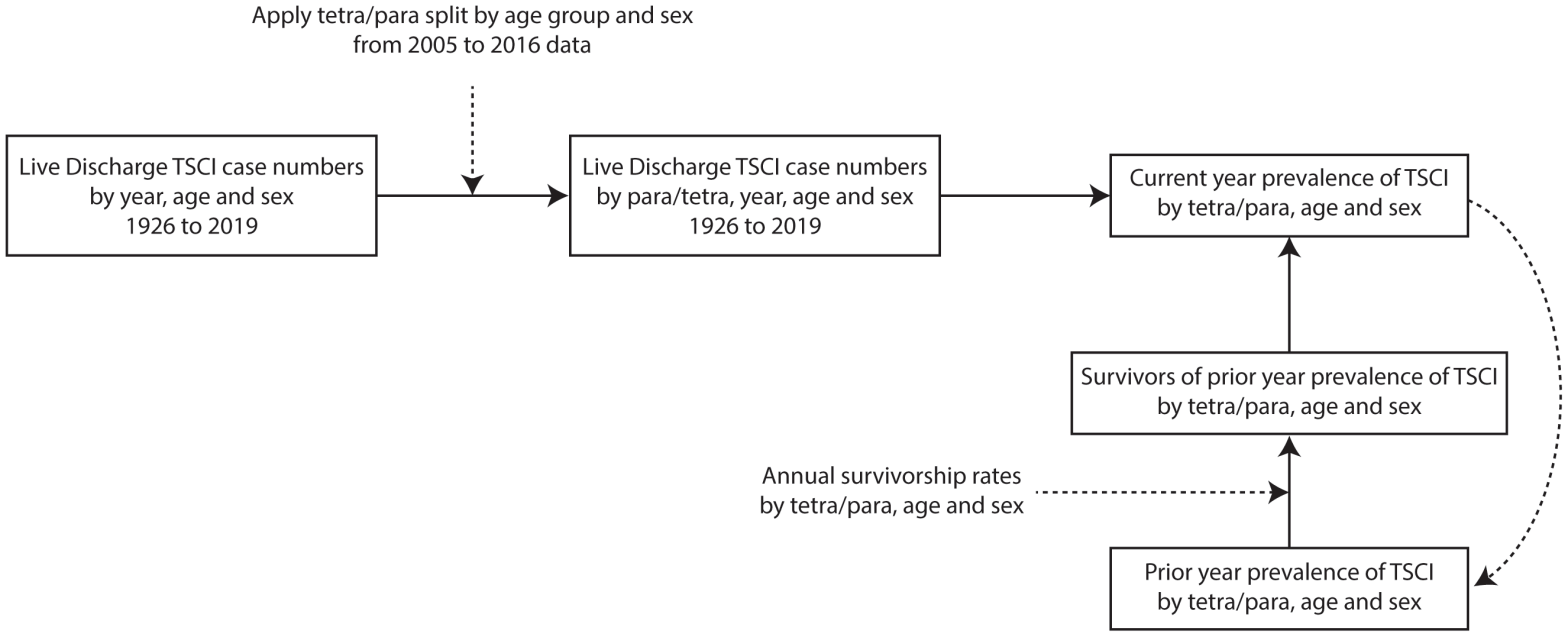
Process map of TSCI prevalence estimation methodology. All TSCI case numbers and rates are calculated by five-year age groups. TSCI, traumatic spinal cord injury; tetra/para, tetraplegia and paraplegia.

The methodology for calculating the prevalence of TSCI in Canada is shown in Figure 2. The age- and sex-specific TSCI discharge incidence rates predicted back to 1926 are required as input data for the prevalence model (see Supplementary Figure 2). The annual discharge case estimates by age were grouped into people with tetraplegia and paraplegia using ICD-10 codes (for estimated percentages see Supplementary Table 3). The annual incidence population for tetraplegia and paraplegia was added to the survivors from previous years in a cohort survival population model (Figure 2). Cohort survivors were calculated by applying annual survivorship rates for tetraplegia and paraplegia derived from data on relative life expectancy (for life expectancy see Supplementary Table 4) (18) to estimate prevalence for the year. All data analysis was performed using SAS version 9.2. The study protocol was approved by the university ethics board.

## Results

### Provincial TSCI incidence rates

Provincial incidence rates based on hospital admission from 2005 to 2016 are shown in Table 2. The province of Ontario had the lowest admission incidence rate (22.7 per million) and Saskatchewan had the highest admission incidence rate (41.3 per million) over the 12-year data range from the health administrative data.

**Table 2:** Provincial traumatic spinal cord injury admission incidence rates per million population, ranked by population size.

Province	2005	2006	2007	2008	2009	2010	2011	2012	2013	2014	2015	2016	Total
Ontario	21.71	20.69	23.11	20.10	18.62	22.46	24.28	23.71	21.98	23.83	24.80	26.62	22.71
British Columbia	34.56	39.37	37.52	38.40	37.18	30.00	32.67	35.85	34.20	42.83	37.49	36.99	36.43
Alberta	30.71	37.70	41.26	39.77	38.60	33.49	32.72	33.50	32.02	31.16	27.53	31.16	33.95
Manitoba	33.10	38.87	36.99	30.89	29.79	35.22	36.47	39.99	27.66	24.98	33.97	37.93	33.80
Saskatchewan	35.23	34.26	46.90	47.18	44.45	39.95	40.32	38.67	38.92	47.29	45.97	35.70	41.26
Nova Scotia	21.32	25.59	27.81	24.58	26.65	31.84	25.41	28.57	34.99	29.72	24.43	31.62	27.72
New Brunswick	32.08	29.51	25.49	24.10	34.67	29.21	30.44	34.36	37.05	35.78	26.53	38.29	31.48
Newfoundland and Labrador	NR	NR	23.57	29.32	27.09	19.16	28.57	28.49	36.03	45.42	35.93	30.17	25.44

Data in this table were derived from the CIHI data request without estimates on Quebec. NR, not reported due to low number of incident cases.

### National Incidence and prevalence rates

For 2019, it was estimated that there were 1,199 cases of TSCI admitted to hospital (32.0 per million), with 123 in-hospital deaths (10% of admissions) and 1,076 cases of TSCI discharged (28.7 per million). The discharges were projected to include 728 cases of tetraplegia (68% of the cases) and 348 cases of paraplegia (32%). The estimated number of people living with TSCI was 30,239 (804/million); 15,533 with paraplegia (52%) and 14,706 with tetraplegia (48%).

Over 15 years, the estimated admission incidence of TSCI in Canada increased by 37% when compared to 2005 when there were 874 cases, with an incidence of 27.1 per million. There was a 134% increase in the number of admissions within the 65+ age group over the 15 year study period, compared to a 9% increase in the <65 age group for the same period. The discharge incidence increased by 41% from 814 cases (25.2 per million) in 2005, with the 65+ age group admissions increasing by 130%, compared to the <65 age group which increased by 9%. The prevalence declined by 5%, from 31,727 cases (984 per million) in 2005. Individuals with tetraplegia were more prevalent (from 64 to 68% of discharge incidence) and had a reduced life expectancy compared to individuals with paraplegia reported in the literature (18). This was incorporated into the prevalence model (see Supplementary Table 4). Additionally, the age at injury increased, thereby impacting survival and prevalence.

**Figure 3:**
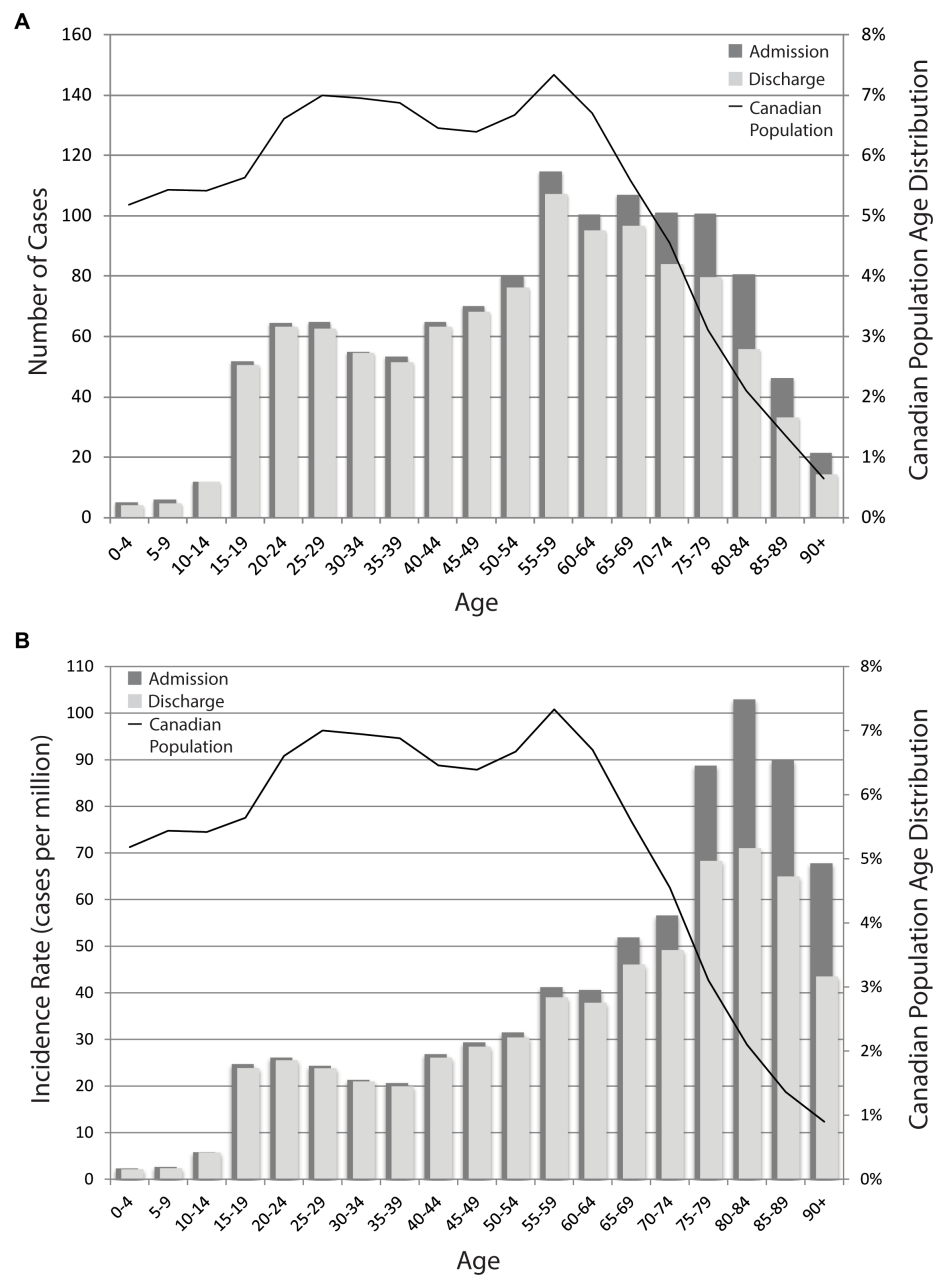
**(A)** Estimated 2019 national TSCI incidence case numbers. Age-specific incidence in 2019, as shown by the number of cases. **(B)** Estimated 2019 national TSCI incidence rates. Age-specific incidence in 2019 as shown by the cases per million per population. Rates are estimated using the case number and the age-specific Canadian population. The bars correspond to the y-axis on the left and the black line corresponds to the y-axis on the right.

**Figure 4:**
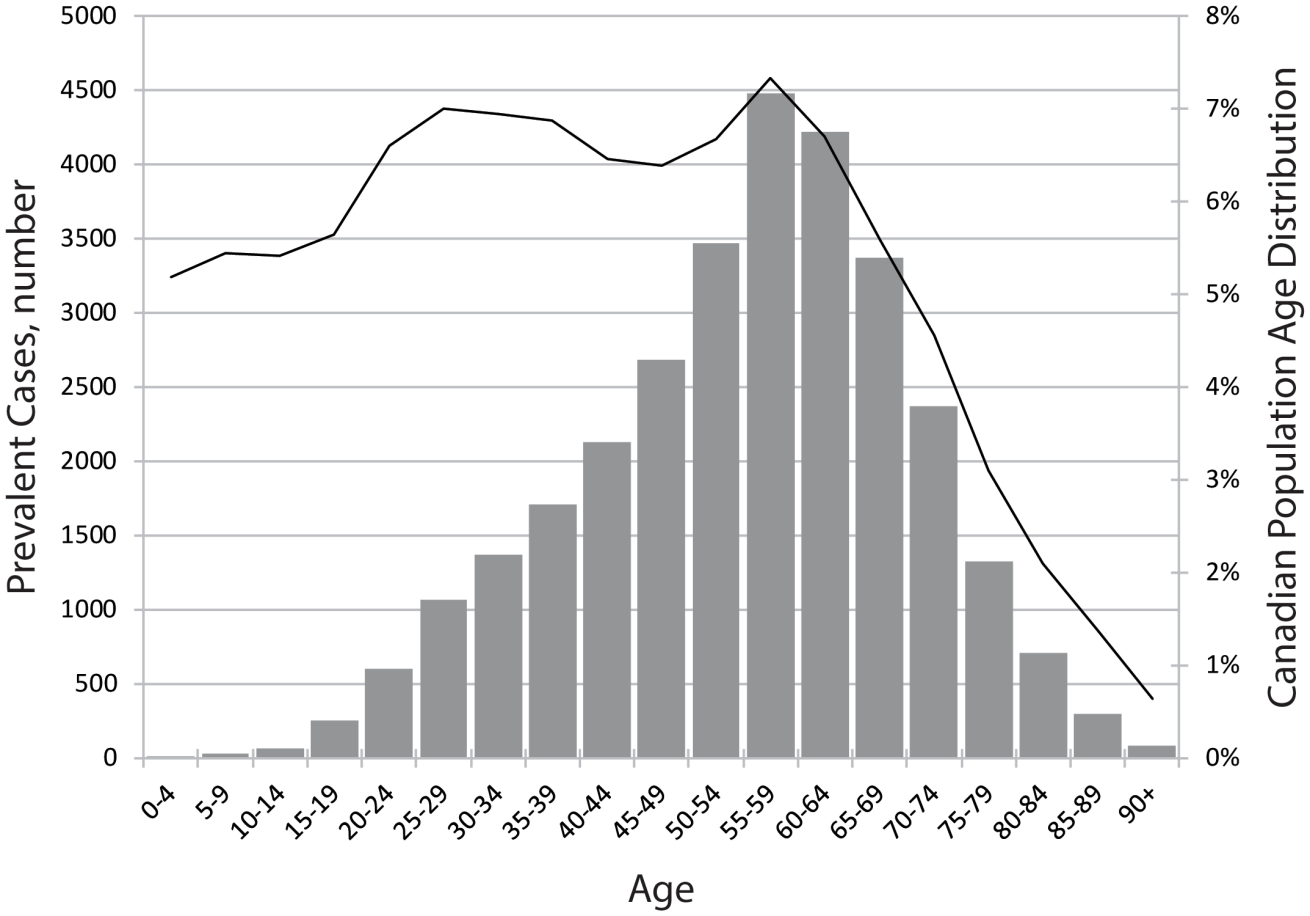
Estimated 2019 national TSCI prevalence numbers. Age-specific prevalence as shown by the number of people. The bars correspond to the y-axis on the left and the black line corresponds to the y-axis on the right.

Estimated age-specific TSCI incidence case numbers are shown in Figure 3A and the resultant age-specific TSCI incidence rates (cases per million) estimated for 2019 are shown in Figure 3B, alongside the age distribution of the Canadian population. Further exploration of estimated 2019 national discharge rates by age- and sex-specific groups are shown in Table 3. The discharge rates are higher for males in almost all of the age groups, except the 0 to 4-year age group. The age-specific prevalence estimates are shown in Figure 4 and the prevalence details on age and injury level are in Table 4. Additional years of admission, discharge and prevalence data going back to 2005 (by five-year age groups and sex) are available upon request.

**Table 3:** National age- and sex-specific live discharge incidence rates of TSCI in Canada for 2019.

Age, years	Male	Female	Total
0–4	2.0	2.3	2.2
5–9	3.9	0.7	2.4
10–14	6.6	4.9	5.7
15–19	33.5	13.8	23.9
20–24	38.1	11.8	25.5
25–29	37.9	8.8	23.8
30–34	33.6	8.1	21.0
35–39	32.0	7.9	19.9
40–44	40.5	12.0	26.1
45–49	47.8	9.3	28.4
50–54	46.4	14.6	30.4
55–59	61.6	16.8	39.1
60–64	58.9	17.6	37.9
65–69	68.8	24.6	46.1
70–74	71.8	28.3	49.2
75–79	82.3	56.0	68.3
80–84	88.4	57.2	71.0
85–89	97.3	43.4	65.0
90+	67.7	32.8	43.5
Total	42.1	15.3	28.6

Incidence rates are shown as cases per million per population.

**Table 4:** Estimated age and severity-specific prevalence of TSCI in Canada for 2019.

Age, years	Paraplegia	Tetraplegia	Total
0–4	4	9	12
5–9	11	18	29
10–14	25	40	65
15–19	113	141	254
20–24	287	316	603
25–29	528	535	1,062
30–34	685	685	1,370
35–39	845	868	1,712
40–44	1,030	1,097	2,127
45–49	1,301	1,385	2,686
50–54	1,689	1,776	3,465
55–59	2,204	2,274	4,478
60–64	2,152	2,069	4,221
65–69	1,805	1,565	3,370
70–74	1,337	1,032	2,370
75–79	787	533	1,321
80–84	446	265	711
85–89	214	87	301
90+	71	11	82
Total	15,533	14,706	30,239

Numbers may not sum to age group total due to rounding.

### Demographics over time

**Figure 5:**
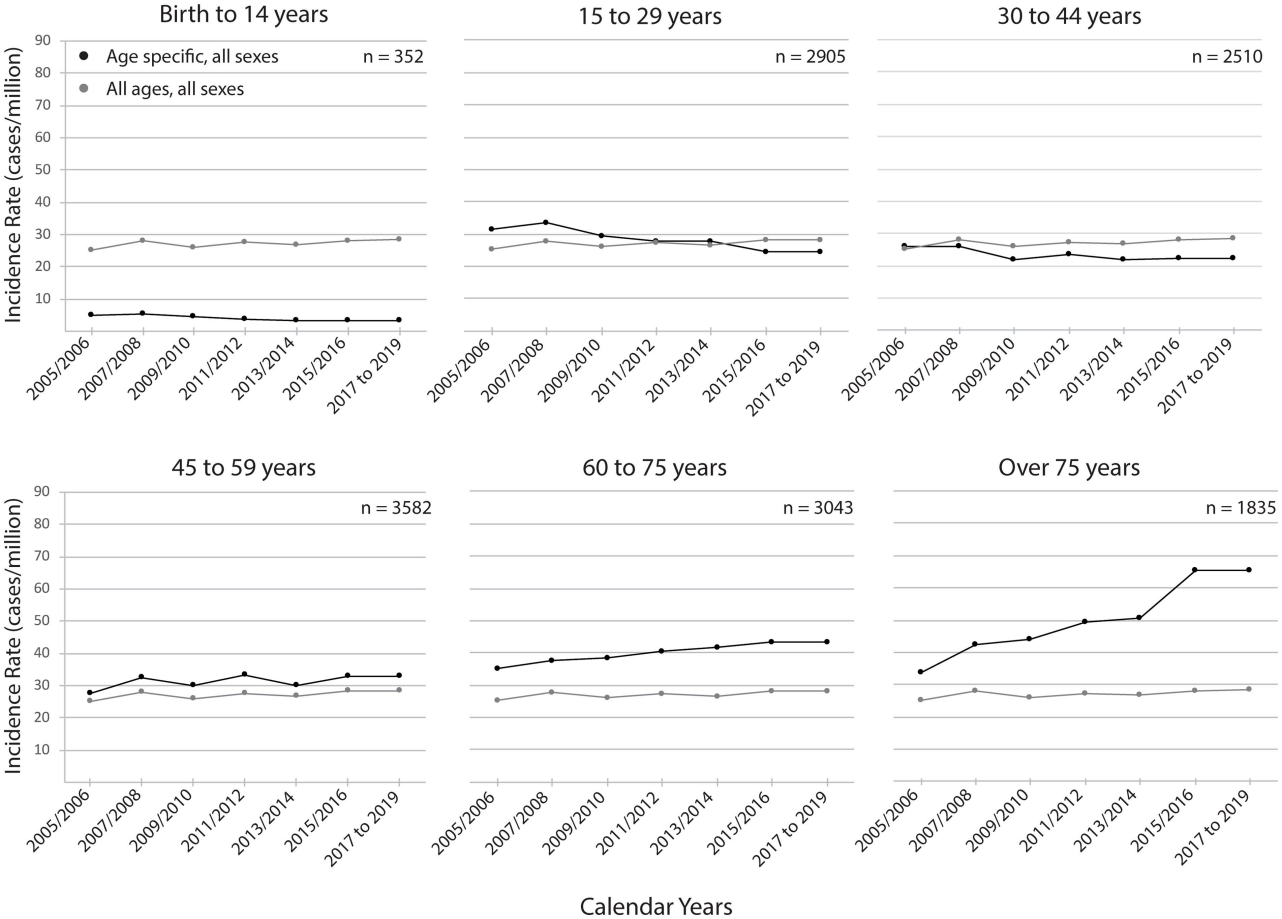
Estimated national TSCI discharge incidence rates, by age group and year. Age-specific discharge incidence rates are shown by cases per million of the age-specific population. The black line represents each age group-specific incidence rates and the gray line represents the incidence rates for all ages as a reference. The 2017 to 2019 age-specific rates were set to 2015 to 2016 rates since that was the most recent year of health administrative data available. The estimated case numbers corresponding to the incidence rates are shown in each panel to provide a context of the estimated case load volume over the 15 calendar years.

Of the estimated incident cases, 76 and 73% were male in 2005 and 2019, respectively. With an increased average age from 46.6 years in 2005 to 54.3 years in 2019; 22% were 65 years or older in 2005 and 38% were 65 years or older in 2019 based on admission incident cases. Data for 15-year age groups are in Figure 5. There are differences in age- and sex-specific discharge incidence rates. In 2005, 52% of the TSCI incidence cases were in individuals under the age of 45, and 48% were 45+; by 2019, the overall incidence rate proportions reversed, with the 45+ group accounting for 66% and the under 45 age group for 34%.

**Figure 6:**
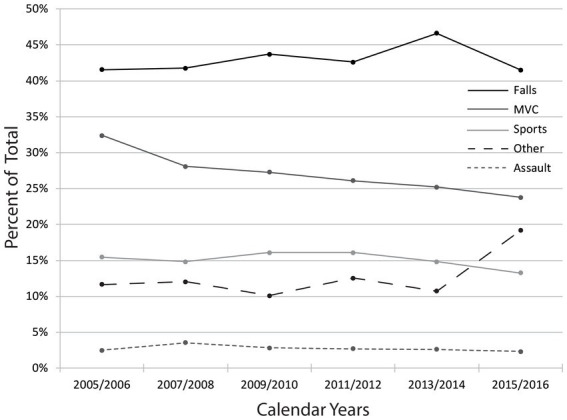
External cause of traumatic spinal cord injury in Canada (excluding Québec), by year. Data are based on the administrative data request and external cause of injury codes. External cause of injury can be viewed by age groups in the Supplementary Table 5.

The external cause of injury for the CIHI data (excluding Québec) is shown in Figure 6. Falls were the most common cause of injury over the 12-year range accounting for 42% followed by motor vehicle collisions (MVC) at 27%. The most common cause varied by age, with sports being the most common for the 0 to 14-year age group (38%), MVC for the 15–29 (42%) and 30–44 age groups (35%), and falls for all other age groups (45–59 age group, 44%; 60–74 age group, 63%; and 75+ age group, 73%). Further breakdown by cause of injury is provided in Supplementary Table 5.

## Discussion

Results of this study estimated that in 2019, there were 1,199 cases of TSCI admitted to acute care hospitals (32.0 per million) and 1,076 cases discharged to the community (28.7 per million) in Canada. The estimated prevalence of TSCI in 2019 was 30, 239 (804/million). Using data from 2005 to 2016 and projecting to 2019 revealed trends including, an increased overall number of people injured each year from 874 to 1,199 incident cases, an older average age at injury rising from 46.6 years to 54.3 years and a larger proportion over the age of 65 changing from 22 to 38% during the 15-year time frame. This work contributes to the literature by using a previously published prevalence model with national-level data and establishing a standard way to report on incidence using health administrative data. Clinicians, health care programs and community partners, must anticipate an increasing number of cases of TSCI, particularly involving an older population. In addition, prevention programs should focus on the elderly and the soon-to-be elderly, in an attempt to reduce the incidence of TSCI in these populations.

Differences in the results between our 2010 estimates and the current estimates can be summarized by three factors: the data sources, case identification and time periods the data cover. First, when assessing the data sources, the geographic coverage, cohort size, in-hospital mortality and life expectancy data should be considered. The current 2019 estimates used data from nine of 10 provinces plus all territories, and a cohort of more than 8,000 cases. Estimated rates were applied to the population of a single province (Québec) since the data were not available. Additionally, age-specific in-hospital mortality and updated life expectancy data were incorporated into the current prevalence model. In 2010, data from the province of Alberta was used as it provided the population estimate, which included a cohort of 450 cases (10). Assumptions were made when a single province’s age- and sex-specific incidence rates were applied to the population of Canada and used a single mortality rate for all ages for the 2010 estimates (13).

Second, while both estimates used ICD coding to identify cases of TSCI, there are several differences in case ascertainment relating to the coding version and timing of data collection. The current estimates used ICD-10 codes, in contrast, the 2010 estimates were based on a set of ICD-9 codes. Third, the time periods differ. The current estimates used data collected over 12 years, from 2005 to 2016 with the age- and sex-specific incidence rates from 2015/2016 held constant to estimate rates for 2017 to 2019. The 2010 estimates used data collected over 3 years from 1997 to 1999 (10) and the average incidence rates from the late 1990s were applied to the 2010 Canadian population (13) assuming that the age- and sex-specific incidence rates remained constant for a decade.

Advantages of the 2019 estimates include using recent record-level data, with more accurate coding, covering a greater geographical area with larger sample size, age-specific in-hospital mortality and updated life expectancy data. The drawback to using national-level health administrative data in the 2019 estimates was the lack of medical record verification; data used in the 2010 study were validated. In this study, we were not able to validate with medical records since the national data request was de-identified.

Few studies have been published on the incidence and prevalence of TSCI in Canada over the past decade. A literature review of the incidence of TSCI summarized the Canadian studies, with our previous work representing the only national estimate (13), and regional estimates ranging from 3.6 to 52.5/million (19). The adult incidence rate in the province of Ontario was reported to be 24/million (9) from 2005 to 2011, which aligns with the previous studies from 2003 to 2006 with a range of 23.1 to 24.2/million (7). These published estimates for Ontario are lower than our national estimate of 32/million but are similar to the provincial rates for Ontario from the national health administrative data (from 2005 to 2016 at 18.6 to 26.6/million), although our data includes pediatric TSCI. The idea that provinces have varying incidences has been suggested and this is the first study to show results by province from a single study using the same time period and data collection methods.

Our updated prevalence of 30,239 (804/million) falls in the global range of 236 to 4,187/million (20). The global map for TSCI, an initiative of the International Spinal Cord Society Prevention Committee, reports estimates worldwide (20) and the current results will provide updated estimates on the incidence, prevalence and data on the etiology of TSCI. The Global Burden of Disease study, the only other source of national-level data, reported a 2016 Canadian incidence of 9,654 (2,500/million) and a prevalence of 324,689 (75,200/million) (21), which is 10 times higher than our 2019 estimates. The discrepancy could be explained by differences in the TSCI definition using ICD codes. The Global Burden of Disease study (21) included additional ICD-10 codes that are not included in our case identification, for example, codes for nerves injuries (for a code comparison see Supplementary Table 6). The large differences between these estimates emphasize the importance of an agreed-upon standard set of codes that define TSCI to share data nationally and internationally.

How trauma is defined also contributes to the coding of SCI. The International Classification of External Causes of Injury considers injuries resulting from iatrogenic causes as traumatic (22), but iatrogenic SCI has not been investigated in Canada. Three different studies have reported a 5–18% frequency of iatrogenic SCI with most resulting from spinal surgery (23–25). In the SCI field, the International SCI Data Set Committee considers iatrogenic causes of SCI as traumatic and acknowledged challenges with this classification (26). Despite the decisions of these groups, the CIHI National Trauma Registry does not include iatrogenic causes of injury and many data holdings do not address this discrepancy.

In terms of the age-specific data and external causes of these injuries, the incidence of TSCI increased in the older age groups and decreased in the younger age groups over the 12 years. While the discharge incidence rate for the 15 to 29 age group fell from 31.5/million in 2005 to 24.4/million by 2019. Over the same period, the rate for the 75+ population almost doubled from 33.9/million to 65.4/million, the highest rate for all age groups. The decrease in MVC injuries and fatalities which is a common cause of TSCI in younger populations (see Supplementary Figure 3) (27), an increase in falls recorded in trauma emergency room admissions (17) as well as the increasing proportion of the Canadian population over the age of 65, may explain some of these trends. An increase in TSCI among older individuals due to falls has been observed in data from a Canadian SCI registry (Rick Hansen Spinal Cord Injury Registry) (28) as well as in other countries, including Ireland, the Netherlands, Norway, Switzerland, Czech Republic, Italy, Spain and Korea (23, 29–35). Furthermore, the New Zealand Ministry of Transport reported a decrease in morbidity and mortality (41, 50%) resulting from MVC over 23 years (1993 to 2016) (36). A Canadian pediatric study reported that hospitalization rates from transport-related causes significantly decreased from 2006 to 2012 and suggested provincial prevention policies and legislation targeting drivers, passengers, cyclists and pedestrians may have been responsible (37). Finally, Spain and the US have reported a decreasing incidence in pediatric TSCI and also suggest a decrease in injuries involving vehicles are a contributing factor (38, 39).

The results from this study involving national TSCI data will be helpful to forecast health care resources. Trends toward an increased age of injury, and possibly different causes of injury, will impact planning, funding, delivery and evaluation of injury prevention and health care. Prevention outreach and research should continue to include all ages but there should be an increased awareness of SCI and its causes among clinicians who treat elderly patients and in assistive-living and long-term care settings. With an aging population, there will be a need to examine issues such as end of life decision making and rehabilitation goals, as well as a need to include gerontology experts on clinical teams.

The challenges of using health administrative data are well documented and result in limitations (40, 41). TSCI is a clinical diagnosis that requires training to classify the level and severity of the neurological injury. The varying types of injuries and resultant loss of function comprise a heterogeneous population with sub-categories that are not always captured by ICD-10 coding. Our group conducted previous work on the validity of ICD-10 coding in TSCI, where a clinical diagnosis was compared with ICD-10 codes that found approximately 11% (42) of confirmed TSCI cases did not have a corresponding ICD-10 code for TSCI (Table 1) and therefore would not be included in the data used for this study. Researchers in Ontario reported that these ICD-10-CA codes have high specificity (true negative rate) and moderate sensitivity (true positive rate), also suggesting persons with TSCI could be missed with ICD coding (43). A validation study in Norway found that ICD-9 coding for TSCI can lead to overestimation (44). Using a combination of seven ICD-10 codes (which corresponds to all except two codes in our list) this group reported that approximately 16% of TSCI patients were missed (44). Based on the published ICD-10 coding validity work, the estimated 1,199 cases admitted to Canadian hospitals in 2019 are conservative and the true number could be approximately 10% higher.

Future work should try and capture SCI cases missed with ICD-10-CA coding by validating records using clinical registries and include SCI cases from iatrogenic causes. Improvements in coding practices and advances in the ICD coding with the introduction of ICD-11 will also assist in capturing more cases of TSCI in health administrative data. Finally, adding data from Québec rather than estimating the incidence, will further enhance the accuracy of these results.

In conclusion, this study reported an updated conservative estimate for the incidence and prevalence of TSCI in Canada using national health administrative data and compared to previous estimates. In Canada, because trends and regional differences in TSCI incidence exist, estimates should be continually updated. To further improve these estimates, work is needed to include incident cases of TSCI not captured by current coding methods in health administrative data. These results have implications for planning health care resources, informing prevention strategies, and establishing research priorities in the elderly who are susceptible to TSCI caused by falls.

## Data availability statement

The original contributions presented in the study are included in the article/Supplementary material, further inquiries can be directed to the corresponding author.

## Ethics statement

Ethical review and approval of this study was completed by the University of British Columbia Research Ethics Board (H15-00471). Written informed consent from the patients/participants or patients/participants’ legal guardian/next of kin was not required to participate in this study in accordance with the national legislation and the institutional requirements.

## Author contributions

NT and VN designed the work and involved with acquisition of the data. NT and XC were responsible for data analysis. NT drafted the work. VN, XC, NF, SH, ND, BK, and MD critically reviewed and revised the work. All authors were involved with reviewing and interpreting the data and approved the final version of the work.

## Funding

This study was supported by funding from Praxis Spinal Cord Institute and Canadian Federal Government.

## Conflict of interest

NT, VN, XC, NF, and SH were employed by Praxis Spinal Cord Institute.

The remaining authors declare that the research was conducted in the absence of any commercial or financial relationships that could be construed as a potential conflict of interest.

## Publisher’s note

All claims expressed in this article are solely those of the authors and do not necessarily represent those of their affiliated organizations, or those of the publisher, the editors and the reviewers. Any product that may be evaluated in this article, or claim that may be made by its manufacturer, is not guaranteed or endorsed by the publisher.

## Supplementary material

SUPPLEMENTARY TABLE 1 External Cause of Injury Codes for Iatrogenic Causes.

SUPPLEMENTARY TABLE 2 Aggregated data from CIHI data request (National Trauma Registry & Discharge Abstract Database) for Canada (except Quebec) of Admission and Discharge Case Number, by Year.

SUPPLEMENTARY TABLE 3 TSCI by Tetraplegia, Paraplegia, Age Group and Sex for 2005 to 2016, CIHI data (excluding Quebec).

SUPPLEMENTARY TABLE 4 Estimated Life Expectancy for Persons Living with TSCI as a Percent of that for their Peers in the General Population.

SUPPLEMENTARY TABLE 5 External Cause of Injury by Age, 2005 to 2016, percent of total.

SUPPLEMENTARY TABLE 6 Comparison of ICD-10 Codes for TSCI.

SUPPLEMENTARY FIGURE 1 CIHI data schematic.

SUPPLEMENTARY FIGURE 2 Detailed process flow (incidence and prevalence).

SUPPLEMENTARY FIGURE 3 MVC injury and fatality crude rates, Canada 1984–2017.
