# Supplementary material for: Incidence and prevalence of traumatic spinal cord injury in Canada using health administrative data
Source: Front Neurol. 2023 Jul 24;14:1201025. doi: 10.3389/fneur.2023.1201025 (PMC10406385; doi:10.3389/fneur.2023.1201025)
Supplement: SUPPLEMENTARY TABLE 1 — External Cause of Injury Codes for Iatrogenic Causes. [file Table_1.docx]

**Supplementary Table 1**. External Cause of Injury Codes for Iatrogenic Causes.

| **Mechanism of Injury Grouping** | **ICD-10 Code** |
| --- | --- |
| Complications, misadventures, adverse incidents/reactions to medical and surgical care | Y40, Y41, Y42, Y43, Y44, Y45, Y46, Y47, Y48, Y49, Y50, Y51, Y52, Y53, Y54, Y55, Y56, Y57, Y58, Y59, Y60, Y61, Y62, Y63, Y64, Y65, Y66, Y69, Y70, Y71, Y72, Y73, Y74, Y75, Y76, Y77, Y78, Y79, Y80, Y81, Y82, Y83, Y84, Y88 |
