# Supplementary material for: Incidence and prevalence of traumatic spinal cord injury in Canada using health administrative data
Source: Front Neurol. 2023 Jul 24;14:1201025. doi: 10.3389/fneur.2023.1201025 (PMC10406385; doi:10.3389/fneur.2023.1201025)
Supplement: SUPPLEMENTARY TABLE 2 — Aggregated data from CIHI data request (National Trauma Registry & Discharge Abstract Database) for Canada (except Quebec) of Admission and Discharge Case Number, by Year. [file Table_2.docx]

**Supplementary Table** **2**. Aggregated data from CIHI data request (National Trauma Registry & Discharge Abstract Database) for Canada (except Quebec) of Admission and Discharge Case Number, by Year.

|  | **Year** | | | | | | | | | | | |
| --- | --- | --- | --- | --- | --- | --- | --- | --- | --- | --- | --- | --- |
|  | **2005** | **2006** | **2007** | **2008** | **2009** | **2010** | **2011** | **2012** | **2013** | **2014** | **2015** | **2016** |
| **Admission Cases** | 644 | 689 | 750 | 713 | 699 | 707 | 745 | 772 | 745 | 822 | 795 | 847 |
| **Died** | 59 | 58 | 53 | 45 | 50 | 63 | 66 | 54 | 62 | 84 | 78 | 81 |
| **Live Discharge Cases** | 585 | 631 | 697 | 668 | 649 | 644 | 679 | 718 | 683 | 738 | 717 | 766 |

Note: These total case counts are summarized here by year only. These case numbers were further divided by age and sex for paired years. Those more specific case numbers are not shown here to adhere to privacy requirements, such that some cell sizes are too small to report.
