# Supplementary material for: Incidence and prevalence of traumatic spinal cord injury in Canada using health administrative data
Source: Front Neurol. 2023 Jul 24;14:1201025. doi: 10.3389/fneur.2023.1201025 (PMC10406385; doi:10.3389/fneur.2023.1201025)

**Supplementary Table 3.** TSCI by Tetraplegia, Paraplegia, Age Group and Sex for 2005 to 2016, CIHI data (excluding Quebec).


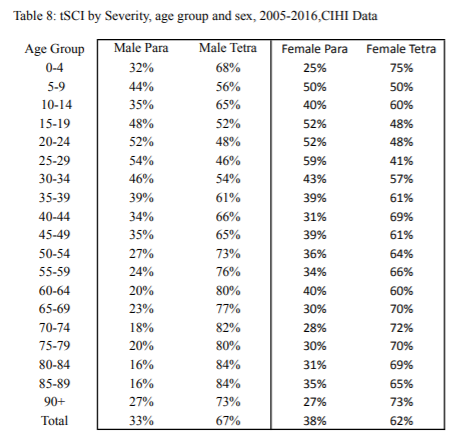

Supplement: SUPPLEMENTARY TABLE 3 — TSCI by Tetraplegia, Paraplegia, Age Group and Sex for 2005 to 2016, CIHI data (excluding Quebec). [file Table_3.docx]
