# Supplementary material for: Incidence and prevalence of traumatic spinal cord injury in Canada using health administrative data
Source: Front Neurol. 2023 Jul 24;14:1201025. doi: 10.3389/fneur.2023.1201025 (PMC10406385; doi:10.3389/fneur.2023.1201025)

**Supplementary Table 4.** Estimated Life Expectancy for Persons Living with TSCI as a Percent of that for their Peers in the General Population.


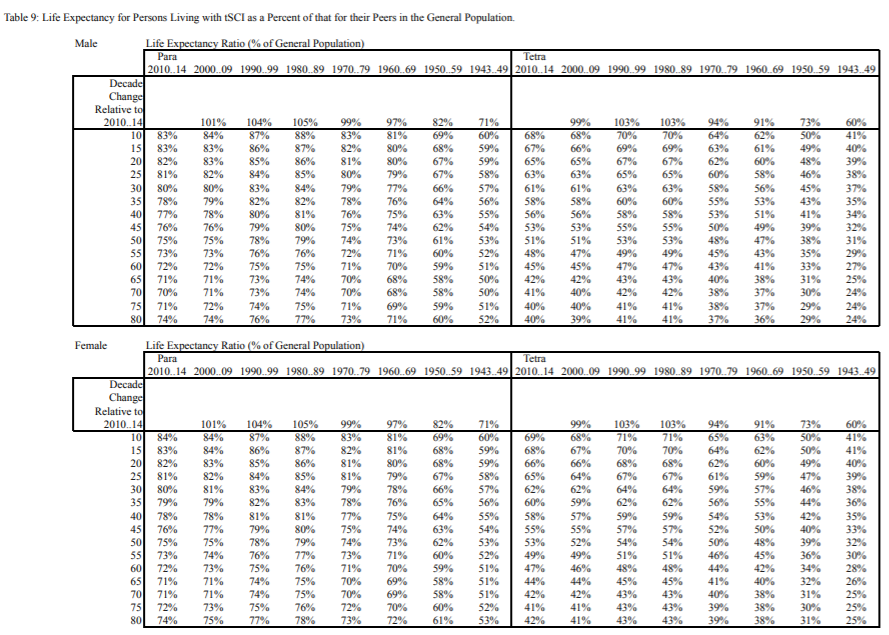

Supplement: SUPPLEMENTARY TABLE 4 — Estimated Life Expectancy for Persons Living with TSCI as a Percent of that for their Peers in the General Population. [file Table_4.docx]
