# Supplementary material for: Incidence and prevalence of traumatic spinal cord injury in Canada using health administrative data
Source: Front Neurol. 2023 Jul 24;14:1201025. doi: 10.3389/fneur.2023.1201025 (PMC10406385; doi:10.3389/fneur.2023.1201025)
Supplement: SUPPLEMENTARY TABLE 5 — External Cause of Injury by Age, 2005 to 2016, percent of total. [file Table_5.docx]

**Supplementary Table 5**. External Cause of Injury by Age, 2005 to 2016, percent of total.

| Cause | Age Group | | | | | |
| --- | --- | --- | --- | --- | --- | --- |
|  | 0 - 14 | 15 - 29 | 30 - 44 | 45 - 59 | 60 - 74 | 75+ |
| Falls | 16% | 16% | 24% | 44% | 63% | 73% |
| MVC | 28% | 42% | 35% | 26% | 17% | 13% |
| Sports | 38% | 24% | 22% | 14% | 7% | 1% |
| Other | 15% | 11% | 14% | 13% | 13% | 13% |
| Assault | 4% | 6% | 4% | 2% | 1% | 0% |
