# Supplementary material for: Incidence and prevalence of traumatic spinal cord injury in Canada using health administrative data
Source: Front Neurol. 2023 Jul 24;14:1201025. doi: 10.3389/fneur.2023.1201025 (PMC10406385; doi:10.3389/fneur.2023.1201025)
Supplement: SUPPLEMENTARY TABLE 6 — Comparison of ICD-10 Codes for TSCI. [file Table_6.docx]

**Supplementary Table 6.** Comparison of ICD-10 Codes for TSCI.

| **ICD-10 Code and Description** | | **Canadian**  **Groups** | **GBD** |
| --- | --- | --- | --- |
| S14 – Injury of nerves and spinal cord at neck level | |  |  |
| S14.0 | Concussion and oedema of cervical spinal cord | X | X |
| S14.1 | Other and unspecified injuries of cervical spinal cord | X* | X |
| S14.2 | Injury of nerve root of cervical spine |  | X |
| S14.3 | Injury of brachial plexus |  | X |
| S14.4 | Injury of peripheral nerves of neck |  | X |
| S14.5 | Injury of cervical sympathetic nerves |  | X |
| S14.8 | Injury of other specified nerves of neck |  | X |
| S14.9 | Injury of unspecified nerves of neck |  | X |
| S24 – Injury of nerves and spinal cord at thoracic level | | | |
| S24.0 | Concussion and oedema of thoracic spinal cord | X | X |
| S24.1 | Other and unspecified injuries of thoracic spinal cord | X* | X |
| S24.2 | Injury of nerve root of thoracic spine |  | X |
| S24.3 | Injury of peripheral nerves of thorax |  | X |
| S24.4 | Injury of thoracic sympathetic nervous system |  | X |
| S24.8 | Injury of other specified nerves of thorax |  | X |
| S24.9 | Injury of unspecified nerves of thorax |  | X |
| S34 – Injury of lumbar and sacral spinal cord and nerves at abdomen, lower back and pelvis level | | | |
| S34.0 | Concussion and oedema of lumbar and sacral spinal cord | X | X |
| S34.1 | Other and unspecified injuries of lumbar and sacral spinal cord | X* | X |
| S34.2 | Injury of nerve root of lumbar and sacral spine |  | X |
| S34.3 | Injury of cauda equina | X | X |
| S34.4 | Injury of lumbosacral plexus |  | X |
| S34.5 | Injury of lumbar, sacral and pelvic sympathetic nerves |  | X |
| S34.6 | Injury of peripheral nerve(s) at abdomen, lower back and pelvis level |  | X |
| S34.8 | Injury of other nerves at abdomen, lower back and pelvis level |  | X |
| S34.9 | Injury of unspecified nerves at abdomen, lower back and pelvis level |  | X |
| T06 – Other injuries involving multiple body regions, not elsewhere classified | | | |
| T06.0 | Injuries of brain and cranial nerves with injuries of nerves and spinal cord at neck level | X |  |
| T06.1 | Injuries of nerves and spinal cord involving other multiple body regions | X |  |
| T91 – Sequelae of injuries of neck and trunk | | | |
| T91.3 | Sequelae of injury of spinal cord |  | X |

*Not all sub-codes of the ICD-10-CA are included in the ‘Canadian’ set of codes used to identify TSCI
