# Supplementary figures and images for: Incidence and prevalence of traumatic spinal cord injury in Canada using health administrative data
Source: Front Neurol. 2023 Jul 24;14:1201025. doi: 10.3389/fneur.2023.1201025 (PMC10406385; doi:10.3389/fneur.2023.1201025)

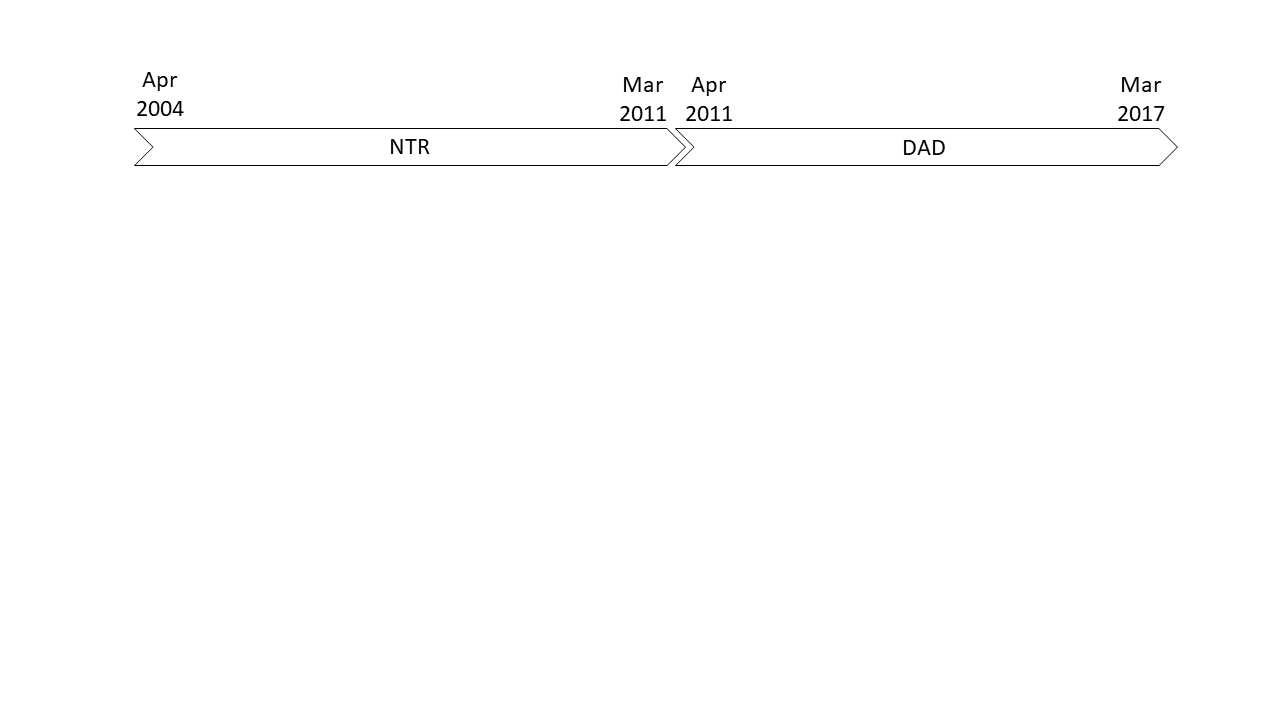

Supplement: SUPPLEMENTARY FIGURE 1 — CIHI data schematic. [file Image_1.tif]

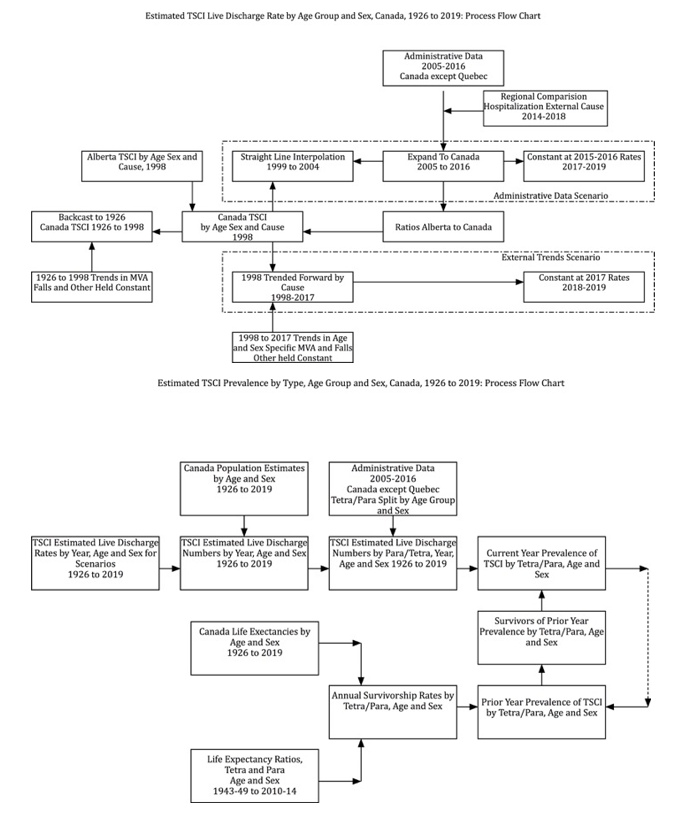

Supplement: SUPPLEMENTARY FIGURE 2 — Detailed process flow (incidence and prevalence). [file Image_2.tif]

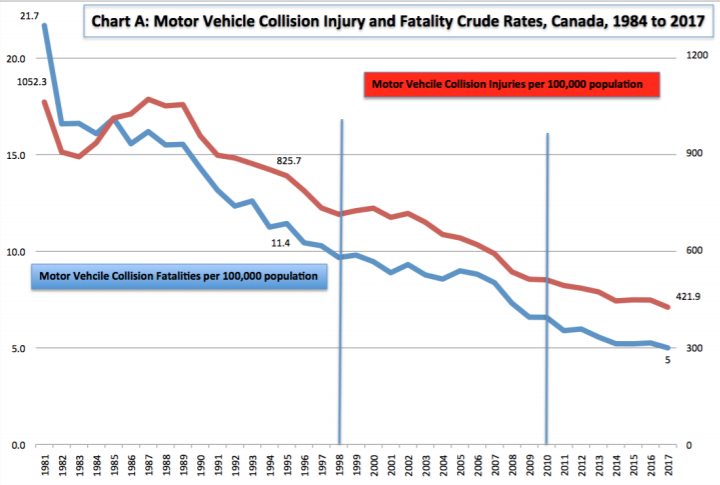

Supplement: SUPPLEMENTARY FIGURE 3 — MVC injury and fatality crude rates, Canada 1984–2017. [file Image_3.tif]
